# Small GTP-binding protein PdRanBP regulates vascular tissue development in poplar

**DOI:** 10.1186/s12863-016-0403-4

**Published:** 2016-06-29

**Authors:** Shaofeng Li, Qinjun Huang, Bingyu Zhang, Jianhui Zhang, Xue Liu, Mengzhu Lu, Zanmin Hu, Changjun Ding, Xiaohua Su

**Affiliations:** Experimental Center of Forestry in North China, Chinese Academy of Forestry, Beijing, 100023 People’s Republic of China; State Key Laboratory of Tree Genetics and Breeding, Research Institute of Forestry, Chinese Academy of Forestry, Key Laboratory of Tree Breeding and Cultivation, State Forestry Administration, Beijing, 100091 People’s Republic of China; Plants for Human Health Institute, Department of Horticultural Science, North Carolina State University, 600 Laureate Way, Kannapolis, North Carolina 28081 USA; Biomarker Technologies Corporation, Beijing, 101300 People’s Republic of China; Institute of Genetics and Developmental Biology, Chinese Academy of Sciences, Beijing, 100101 People’s Republic of China

**Keywords:** Molecular cloning, Functional analysis, *PdRanBP*, *Populus deltoides*, Vascular tissue

## Abstract

**Background:**

Previous research has demonstrated that ectopic expression of Ran-binding protein (RanBP) in *Arabidopsis* results in more axillary buds and reduced apical dominance compared to WT plants. However, the function of *RanBP* in poplar, which has very typical secondary growth, remains unclear. Here, the *Populus deltoides* (Marsh.) *RanBP* gene (*PdRanBP*) was isolated and functionally characterized by ectopic expression in a hybrid poplar (*P. davidiana* Dode × *P. bolleana* Lauche).

**Results:**

*PdRanBP* was predominantly expressed in leaf buds and tissues undergoing secondary wall expansion, including immature xylem and immature phloem in the stem. Overexpression of *PdRanBP* in poplar increased the number of sylleptic branches and the proportion of cells in the G2 phase of the cell cycle, retarded plant growth, consistently decreased the size of the secondary xylem and secondary phloem zones, and reduced the expression levels of cell wall biosynthesis genes. The downregulation of *PdRanBP* facilitated secondary wall expansion and increased stem height, the sizes of the xylem and phloem zones, and the expression levels of cell wall biosynthesis genes.

**Conclusions:**

These results suggest that *PdRanBP* influences the apical and radial growth of poplar trees and that *PdRanBP* may regulate cell division during cell cycle progression. Taken together, our results demonstrated that PdRanBP is a nuclear, vascular tissue development-associated protein in *P. deltoides*.

**Electronic supplementary material:**

The online version of this article (doi:10.1186/s12863-016-0403-4) contains supplementary material, which is available to authorized users.

## Background

Forests provide the raw materials for a very large amount of wood products. The process of wood formation and development is mediated by the activity of the vascular cambium, which is a meristematic cell population that facilitates vascular tissues development in tree stems [1]. The development of vascular tissue (secondary xylem and secondary phloem) includes the emergence of new tissues through regular cell division, horizontal and radial extension and, ultimately, cell maturation [2–4]. However, the mechanisms that regulate secondary wall thickening and subsequent expansion of the stems remain largely unknown. Genetic engineering could be used to improve specific traits in plants without the need for long-term breeding, and other valuable traits can be stably inherited from the parental genetic material [5–9].

Small GTP-binding genes play diverse roles in a multitude of cellular processes, such as microtubule organization, vesicle-mediated intracellular trafficking, signal transduction, and cell growth and division in plants and animals [10, 11]. The Ras-related nuclear protein (Ran or RAN) is a member of an important family of small GTP-binding proteins. Ran interacts with importin or exportin proteins to regulate a variety of biochemical processes, including nuclear envelope assembly, nucleo-cytoplasmic signal transfer, cell cycle progression, light signalling, resistance to pathogens, and the regulation of hormone sensitivities [12–17]. Ran-binding protein (RanBP) is vital for the transit of nuclear proteins between the stages of mitosis and interphase. Lee et al. [18] found that expression of the pea (*Pisum sativum* L., cv. Alaska) *Ran* gene (*PsRan1*) is regulated by various light sources via a phytochrome-mediated signalling pathway. Overexpression of the wheat (*Triticum aestivum* L.) *RAN* gene (*TaRAN1*) increased the amount of primordial tissue, reduced the number of lateral roots, and stimulated hypersensitivity to exogenous auxin in *Arabidopsis thaliana* (L.) and rice (*Oryza sativa* L.) [19]. Virus-induced gene silencing (VIGS) of the *Nicotiana benthamiana* (Domin.) *RanBP* gene (*NbRanBP1*) caused leaf yellowing, abnormal leaf morphology, and stunted growth in transgenic *N. benthamiana* plants. Defence-related genes were induced and mitochondrial membrane potential was reduced in NbRanBP1 VIGS plants [20]. Transgenic *Arabidopsis* expressing the antisense *Arabidopsis RanBP1c* gene (*AtRanBP1c*) displayed enhanced primary root growth but suppressed lateral root growth. Antisense *AtRanBP1c* transgenic plants were hypersensitive to auxin and had an increased mitotic index in both the lateral and primary roots [21]. The overexpression of the *O. sativa RAN* gene (*OsRAN2*) resulted in extreme sensitivity to abscisic acid (ABA), osmotic stress, and salinity in rice and *A. thaliana* [22].

Molecular and genetic studies in tree species (e.g., poplar and *Eucalyptus gunnii*) and *Arabidopsis* have uncovered a number of wood-associated transcription factors and other proteins that might be involved in secondary wall formation [23–25]. Among the identified transcription factors, the best-characterized are the NAC and MYB families. *Populus trichocarpa* (Torr. & Gray) wood-associated NAC domain transcription factors (PtrWNDs) are master switches that activate a suite of downstream transcription factors, such as PtrNAC150, PtrNAC156, PtrNAC157, PtrMYB90, PtrMYB18, PtrMYB74, PtrMYB75, PtrMYB121 and PtrMYB128. These proteins are involved in the coordinated regulation of secondary wall biosynthesis during wood formation [26]. *P. deltoides PdMYB221* has been shown to be involved in the negative regulation of secondary wall formation through the direct and indirect suppression of gene expression related to secondary wall biosynthesis [27]. It has recently been shown that *P. tomentosa PtoMYB92* activates the lignin biosynthetic pathway; specifically, this factor activates the expression of the lignin biosynthetic genes *CCOAOMT1*, *CCR2* and *C3H3* by binding to their promoters [28]. *Eucalyptus gunnii* (J.T. Hook) cinnamoyl coenzyme A reductase (*EgCCR*) is expressed in all lignifying cells (vessel elements and xylem fibres) of xylem tissues and is associated with primary and secondary xylem formation in *Arabidopsis thaliana* [29]. Coleman et al. [30] showed that overexpression of the *Gossypium hirsutum* sucrose synthase gene (*GhSuSy*) in hybrid poplar (*Populus alba* L. × *Populus grandidentata* Michx.) induced thicker cell walls and greater wood density. Furthermore, a recent study in Chinese white poplar (*Populus tomentosa* Carr.) showed that genes associated with lignin biosynthesis, including 4-coumarate:cinnamate-4-hydroxylase(*C4H*), cinnamyl alcohol dehydrogenase(*CAD*), and caffeoyl CoA 3-O-methyltransferase (*CCoAOMT*), were transcribed in the lignified xylem [31]. These studies have significantly improved our understanding of secondary xylem differentiation and secondary wall formation.

*Populus deltoides* (Marsh.), which is widely distributed between the northern latitudes of 40° to 60° in North America, was introduced into China in 1972. This tree is a black poplar tree of the *Aigeiros* section in the *Populus* genus, exhibiting good quality, high yield, disease resistance and strong adaptability. Therefore, *P. deltoides* is widely used as an important species for poplar breeding. However, compared with our understanding of the function of the small GTP-binding protein in *Arabidopsis*, *N. benthamiana*, *O. sativa* and other plants, the functions of small GTP-binding protein genes in tree species remain largely unknown.

In this study, we isolated the *P. deltoides* small GTP-binding protein gene (*PdRanBP*), and observed its expression primarily in leaf buds as well as in immature xylem and immature phloem in the stem. Additionally, the downregulation of *PdRanBP* promoted vegetative growth in poplar. Interestingly, the overexpression of *PdRanBP* induced the formation of sylleptic branches and reduced apical dominancy in hybrid poplar plantlets. This study provides new data that will help to determine the molecular mechanism of *PdRanBP* in *P. deltoides* growth and vascular tissue development.

## Results

### Isolation and phylogenetic analysis of *PdRanBP*

**Fig. 1:**
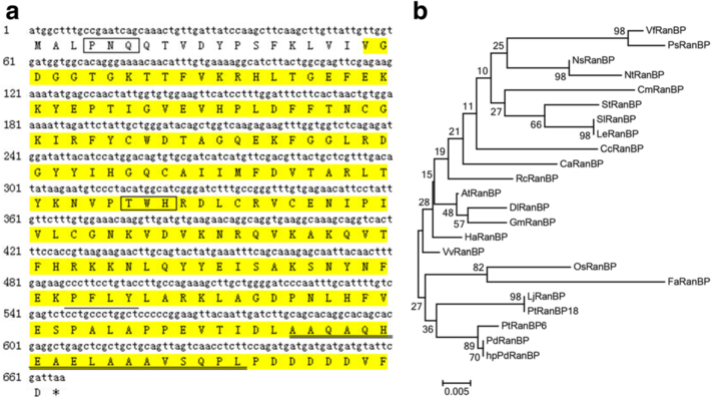
Characterization of the PdRanBP protein. **a** Nucleotide and deduced amino acid sequence of the coding region (cDNA) of PdRanBP from *Populus deltoides*. The highlighted yellow sequence indicates the conserved RAN subfamily domain. The 2Fe-2S ferredoxin-type iron-sulphur-binding domains are boxed. The EGF-like domain is underlined with a thin black line. The double-underlined region is the VWFC domain. The PdRanBP protein sequence was analysed and characterized using the SMART analysis service (http://smart.embl-heidelberg.de/). **b** A neighbor- joining phylogenetic tree was constructed based on an alignment of the protein sequences of AtRanBP [GenBank: AY116939.1], CaRanBP [GenBank: AJ299064.1], CcRanBP [GenBank: AB372270.1], CmRanBP [GenBank: AB015288.1], DlRanBP [GenBank: JF461291.1], FaRanBP [GenBank: FJ610236.1], GmRanBP [GenBank: AK243772.1], HaRanBP [GenBank: AF495716.1], LeRanBP [GenBank: L28714.1], LjRanBP [GenBank: Z73959.1], NtRanBP [GenBank: L16787.1], NsRanBP [GenBank: AY563049.1], OsRanBP [GenBank: AB015288.1], PsRanBP [GenBank: EF194277.1], PtRanBP6 [GenBank: XM_002308612.1], PtRanBP18 [GenBank: XM_002324810.2], RcRanBP [GenBank: XM_002515509.1], SlRanBP [GenBank: NM_001247091.1], StRanBP [GenBank: DQ222522.1], VfRanBP [GenBank: Z24678.1], VvRanBP [GenBank: FQ396597.1], hpPdRanBP [KU841447] and PdRanBP [KU841446]. MEGA 4.0 software was used for this alignment. The 23 RanBP amino acid sequences are shown in Additional file 2. The scale bar indicates the distance calculated using the multiple alignment

A 670-bp cDNA fragment of *P. deltoides RanBP* was amplified by reverse transcription PCR (RT-PCR) and sequenced. The gene from which the cDNA was derived was named *PdRanBP* (Fig. 1a). *PdRanBP* shares 99, 89 and 100 % sequence identity with the open reading frames (ORFs) of *P. trichocarpa RanBP6* (*PtRanBP6*), *PtRanBP18* and *hpPdRanBP* of hybrid poplar (*P. davidiana* Dode × *P. bolleana* Lauche) (Additional file 1), respectively. PdRanBP encodes a polypeptide that is predicted to contain 221 amino acids and to have a molecular weight of 25033.5 Da and an isoelectric point (pI) of 6.38 (Fig. 1a).

Nucleotide sequence analysis revealed that the *PdRanBP* cDNA sequence has 82–100 % similarity to the *RanBP* cDNA sequences from twenty-two other plant species. Phylogenetic analysis (Fig. 1b) of the RanBP amino acid sequences derived from *P. deltoides* and the twenty-two other plant species showed that PdRanBP clusters closely with hybrid poplar (*P. davidiana* Dode × *P. bolleana* Lauche) hpPdRanBP and *P. trichocarpa* PtRanBP6. In addition, the PdRanBP amino acid sequence has 100 and 99.0 % similarity (Additional file 2) to the hpPdRanBP and PtRanBP6 sequences, respectively. We found that PdRanBP contains a core domain between residues 19 and 220 that is structurally similar to the GTP-binding domains of other small GTPases. Based on these findings, PdRanBP is a conserved member of the Ras superfamily of small GTPases. In addition, three other important domains were identified in PdRanBP. Two 2Fe-2S ferredoxin-type iron-sulphur binding domains may exist between residues 4 and 6, and between residues 106 and 108; the conserved cysteine residues of these domains are important elements of various metabolic enzymes. An epidermal growth factor (EGF)-like domain signature was identified in N-terminal half of the PdRanBP protein (between residues 163 and 166). These domains bind to specific cell-surface receptors with a high affinity and induce their dimerization. This event is essential for the activation of tyrosine kinases and the initiation of a signal transduction cascade that results in DNA synthesis and cell proliferation. A von Willebrand factor type C (VWFC) domain is located at the N-terminus (between residues 195 and 212) of PdRanBP; this domain is thought to participate in oligomerization (but not the initial dimerization step) during the formation of large protein complexes (Fig. 1a and Additional file 3).

### The expression pattern of *PdRanBP* in different organs and tissues in *P. deltoides*

**Fig. 2:**
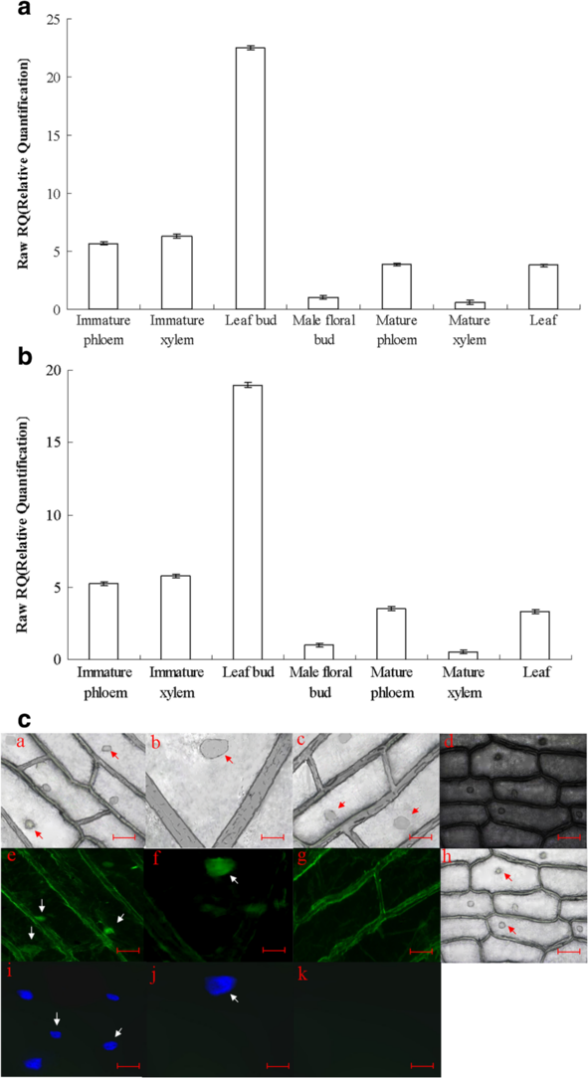
qRT-PCR analysis of the expression of *PdRanBP* in different vascular tissues and organs of *P. deltoides*, and detection of immediate and stable expression of *GFP*-tagged *PdRanBP*. **a**, **b** qRT-PCR analysis of *PdRanBP* expression in the vascular tissues and other organs of *P. deltoides* during secondary cell wall development. Aliquots of 1000 ng total RNA were reverse-transcribed into cDNA. The signals were normalized to the constitutively expressed poplar α-tubulin (*TUA1*) (**a**) and Ubiquitin (*UBQ1*) (**b**) genes. The values are the mean ± standard error (SE) of three replicates. *PdRanBP* was predominantly expressed in the leaf buds, immature xylem and immature phloem of *P. deltoides*. **c** Nuclear localization of EGFP-PdRanBP fusion protein in onion epidermal cells. Dark-field images were captured for green fluorescence (*e* and *f*), GFP-only control (g) and the corresponding bright-field images for *e*, *f, g* are *a, b, c*. Bright-field images (*h*) were captured for cell morphology, and the corresponding dark-field images for *h* is *d*. *i* and *j*, Nuclei counterstained with 4′, 6-diamidino-2-phenylindole (DAPI); the corresponding GFP-only control images of *i* and *j* are shown in *k*. The scale bars are 200 μm in *a*, *c, d, e, g, h, i* and *k*, and 800 μm in *b, f*, and *j*

The *PdRanBP* gene showed highly divergent expression patterns in the tissues tested. Compared with the floral buds, *PdRanBP* was expressed at 3.9- to 6.3-fold higher levels in the immature xylem, immature phloem and mature phloem. However, only weak expression of *PdRanBP* was detected in the mature xylem (Fig. 2a). The observed patterns of gene expression were the same when using either *TUA1* (Fig. 2a) or *UBQ1* (Fig. 2b) as the control gene. *PdRanBP* was predominantly expressed in leaf buds and in the immature xylem and immature phloem, indicating that *PdRanBP* expression correlates with leaf bud development and wood formation in *P. deltoides*.

### Detection of immediate and stable expression of *GFP*-tagged *PdRanBP*

Using gene gun technology, nuclear localization of GFP-tagged PdRanBP was observed in transient expression conditions in onion cells (Fig. 2c, white arrows in panels e and f) and stable expression conditions in poplar stem cells (Fig. 2c, white arrows in panels i and j). Control cells (g and k) did not exhibit any green fluorescence (g) or 4′,6-diamidino-2-phenylindol (DAPI) staining (k) at the settings at which the images were collected.

### Generation of *PdRanBP*-overexpressing and *PdRanBP*-downregulated poplar lines

**Fig. 3:**
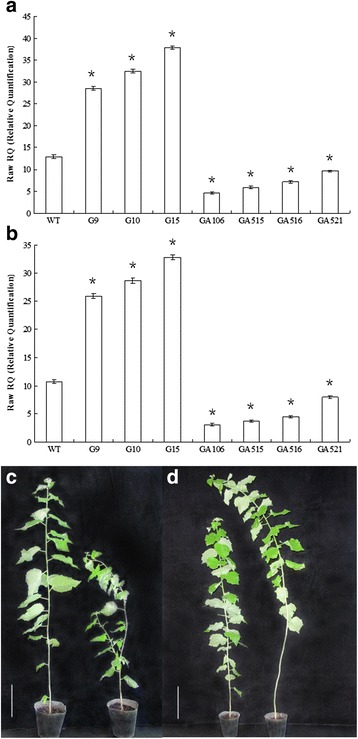
Overexpression of *PdRanBP* stunted growth and induced sylleptic branches; *PdRanBP* downregulation produced taller plants with thicker stems. **a**, **b** qRT-PCR analysis confirming *PdRanBP* overexpression in the transgenic poplar lines G9 and G15. *TUA1* (**a**) and *UBQ1* (**b**) were used as control genes. The error bars represent the standard error (SE) of three replicates. WT: wild poplar; G9, G10 and G15: transgenic poplar lines overexpressing *PdRanBP* (OE); GA106, GA515, GA516 and GA521: transgenic poplar lines in which *PdRanBP* is downregulated (DR). The asterisks indicate significant differences between the transgenic lines and WT (*P < 0.05). **c** Phenotypic comparison of 120-day-old WT poplar (left) and the *PdRanBP*-OE line G15 (right). **d** Phenotypic comparison of 120-day-old WT plants (left) and the *PdRanBP*-DR line GA106 (right). The scale bars correspond to 20.76 cm in B, and 23.8 cm in C

*PdRanBP*-overexpressing (OE) and *PdRanBP*-downregulated (DR) poplar lines (*PdRanBP* antisense lines) were initially screened by PCR amplification of the *NptII*-specific sequence. Six lines (of the 60 independent lines subjected to PCR detection) with high or low level expression of *PdRanBP* were selected for further characterization. Compared with the wild-type (WT) plants, *PdRanBP* was upregulated by 120.82, 150.88 and 192.41 % in the *PdRanBP*-OE lines G9, G10 and G15, respectively (*P* = 0.000, Fig. 3a). *PdRanBP* expression in four independent *PdRanBP*-DR lines (GA106, GA515, GA516 and GA521) was reduced by 64.46, 50, 44.61, and 25 %, respectively, compared with the WT condition (*P* = 0.007, Fig. 3a). The same patterns of gene expression were observed using either *TUA1* (Fig. 3a) or *UBQ1* (Fig. 3b) as the control gene.. These selected transgenic and WT plants were multiplied clonally in vitro, and five plants from each poplar line were cultivated in soil in the greenhouse until a growth age of 120 days.

### *PdRanBP* overexpression causes slow growth and induces sylleptic branches in hybrid poplar

**Fig. 4:**
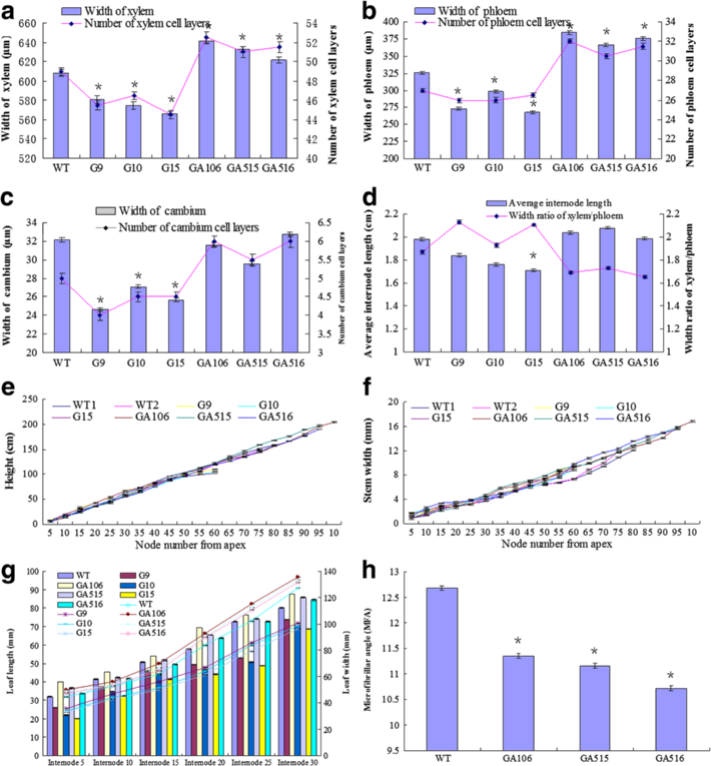
Anatomical features of the stem, plant height, stem width, leaf size and microfibril angle in 120-day-old WT and transgenic poplars expressing sense or antisense *PdRanBP*. For the morphological analyses, the stem base diameter was measured 5 cm above the soil surface using digital callipers. The values are the mean ± standard error (SE) of measurements from three plants. The asterisks indicate significant differences from the wild-type (*P < 0.05). WT: wild-type poplar; WT1: control of *PdRanBP*-OE poplar; WT2: control for the *PdRanBP*-DR poplar; G9, G10 and G15: *PdRanBP*-OE poplar lines; GA106, GA515 and GA516: *PdRanBP*-DR poplar lines. **a** The width of the xylem (left panel) and number of xylem cell layers (right panel) in *PdRanBP*-OE, *PdRanBP*-DR and control lines. The measurements were made at the 15th internode. **b** The width of the phloem (left panel) and number of phloem cell layers (right panel) in the *PdRanBP*-OE, *PdRanBP*-DR and control lines. **c** The width of the cambium (left panel) and number of cambium cell layers (right panel) in the *PdRanBP*-OE, *PdRanBP*-DR and control lines. **d** The average internode length (left panel) and the xylem:phloem width ratio (right panel) in *PdRanBP*-OE, *PdRanBP*-DR and control lines. The average internode length (cm) was calculated by dividing the total height by the total number of nodes. **e**, **f** The *PdRanBP*-DR lines were taller and had wider stems at every fifth node from the apex. **g** A comparison of leaf size in 120-day-old transgenic poplars. Leaf length (left panel) and width (right panel) measurements were performed at the 5th to 30th nodes to compare fully expanded leaves. The values are the mean ± standard error (SE) of 10 leaves. (H) Analysis of the microfibril angle (MFA) in *PdRanBP* transgenic poplar plants. The *PdRanBP*-DR poplar lines had lower MFAs than the control line

**Fig. 5:**
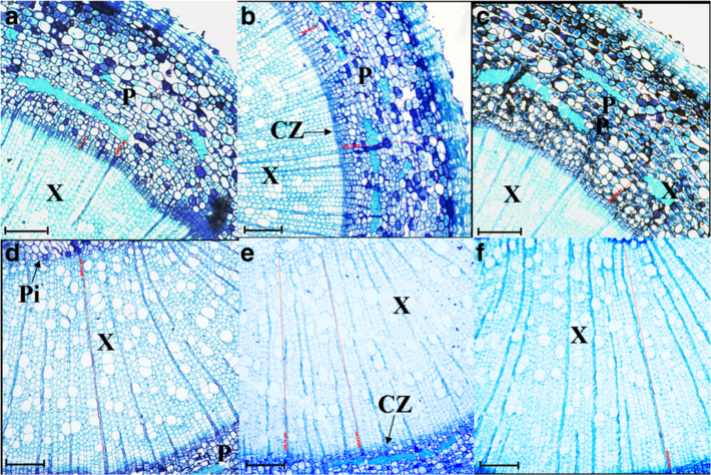
Transverse view of the anatomical features in WT and transgenic poplars expressing sense or antisense *PdRanBP*. The 15th internodes of 120-day-old plants were used to examine the secondary wall cell layers and width of the vascular tissues (**a**, **b**, **c**, **d**, **e** and **f**). Transverse anatomical structure of the stem of a wild-type (WT) poplar (**a**, **d**), *PdRanBP*-OE transgenic poplar (**b**, **e**) and *PdRanBP*-DR transgenic poplar (**c**, **f**). CZ, cambial zone; P, secondary phloem; Pi, pith; C, cortex cells; X, secondary xylem. Scale bars = 100 μm

Three *PdRanBP*-OE lines with high levels of transgene expression were selected for in vitro growth monitoring and phenotypic analyses. Growth was retarded and sylleptic branches were observed in *PdRanBP*-OE plantlets after 120 days of growth in pots; this effect was particularly pronounced in *PdRanBP*-OE line G15 (Fig. 3c). The stem growth of *PdRanBP*-OE poplars was reduced, with a mean decrease in plant height of 44.75 % compared with WT plants (Fig. 4e). A decrease in stem width and average internode length, as well as inhibition of leaf development, were observed in *PdRanBP*-OE lines (Figs. 3c and 4d, f, g). In addition, significant decreases in the width and number of cell layers in the xylem and phloem zones were detected in *PdRanBP*-OE lines compared with WT plants (Figs. 4a, b and 5a, b, d, e). The width and number of cell layers in the cambium were also decreased in *PdRanBP*-OE plants (Fig. 4c). Relative-quantitative real-time PCR (qRT-PCR) and phenotypic data analysis showed that the slow growth correlated positively with the expression level of *PdRanBP*. These experiments demonstrated that increased levels of *PdRanBP* blocked secondary wall synthesis and led to defects in secondary wall expansion.

### *PdRanBP* downregulation promotes growth and facilitates secondary wall expansion

Consistent with the in vitro observations, soil-grown *PdRanBP*-DR GA106, GA515 and GA516 plants were larger overall and showed increased shoot growth compared with WT plants (Figs. 3d and 4e). Increases were also observed in leaf size (leaf length and leaf width), stem diameter, the number of internodes, and average internode length in *PdRanBP*-DR plants (Fig. 4d, f, g). Compared to WT plants, increases in the width and number of cell layers were detected in cross-sections of the primary-secondary transition zone in the stems of transgenic hybrid poplar compared with the WT (every internode from 3th to 7th joint, data not shown). However, these changes were much more pronounced in the stem sections of wood-producing stem tissues (15th internode; Figs. 4a, b, c and 5a, c, d, f). For example, the areas of xylem and phloem in the stele; the number of cell layers in the xylem and phloem; and the xylem, phloem and cambium widths were all increased by the downregulation of *PdRanBP* (Fig. 4a, b, c and Additional file 4).

### The microfibril angle is clearly altered in *PdRanBP*-DR poplar

The microfibril angle (MFA) values in *PdRanBP*-DR poplar lines were 10.49 %–15.46 % lower than that in WT plants, and these differences were statistically significant (*P* = 0.000, Fig. 4h). Thus, *PdRanBP* is likely to be a valuable gene for improving timber strength (i.e., stiffness) in trees.

### Verification of primer specificity and gene-specific PCR amplification efficiency

Ten secondary wall-associated genes encoding transcription factors and other proteins and two reference genes from *P. deltoides* were selected to verify primer specificity and amplification efficiency. The gene name, accession number, gene description, primer sequences, amplification efficiency and correlation coefficients are listed in Additional file 5. The melting temperatures (Tm) of all PCR products ranged from 76.32 °C for *PtrFRA1* to 84.83 °C for *PtrCAD10* (Additional file 6). The amplification efficiency (E) of the PCR reactions varied from 91.29 % for *PtrGT8* to 100.005 % for *PtrCCoAOMT1*, and the correlation coefficients (R^2^) ranged from 0.9933 for *PtrC4H1* and 0.9995 for *PtrGT8* (Additional files 5 and 7).

### *PdRanBP* overexpression and downregulation alter the expression of secondary wall-associated genes

**Fig. 6:**
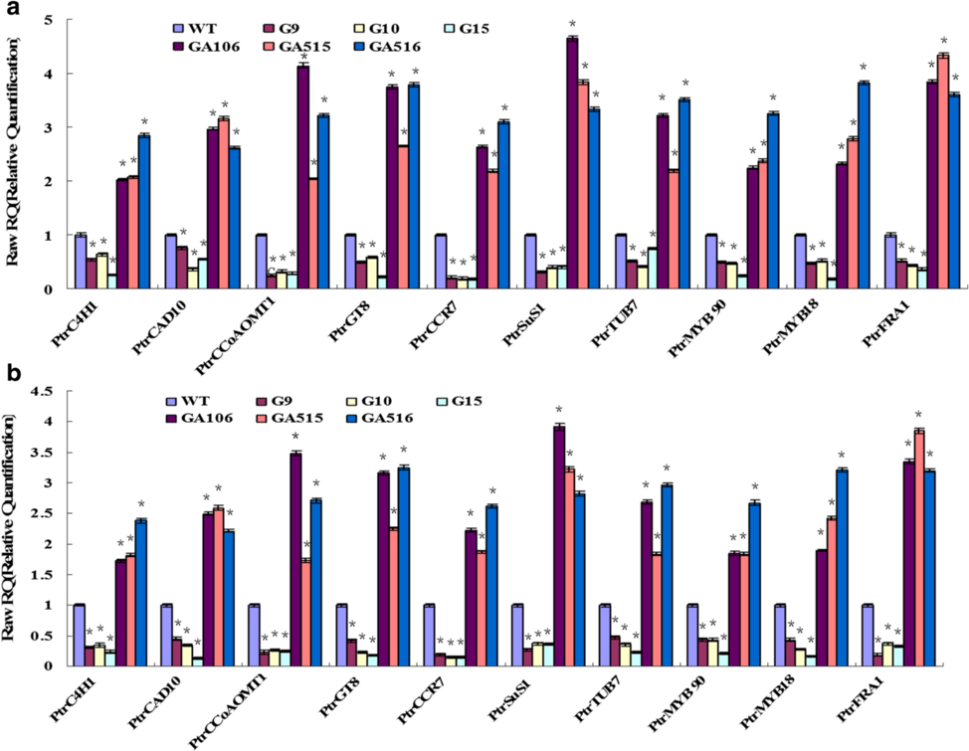
qRT-PCR analysis of secondary cell wall-associated gene expression in *PdRanBP*-OE, *PdRanBP*-DR and WT seedlings. Total RNA was isolated from three biological replicates of the wild-type (WT), *PdRanBP*-OE and *PdRanBP*-DR plants. The expression of trans-cinnamate 4-hydroxylase 1 (*PtrC4H1*), cinnamyl alcohol dehydrogenase 10 (*PtrCAD10*), caffeoyl CoA 3-O-methyltransferase 1 (*PtrCCoAOMT1*), glycosyltransferase 8 (*PtrGT8*), cinnamoyl coenzyme A reductase 7 (*PtrCCR7*), sucrose synthase 1 (*PtrSuS1*), beta-tubulin 7 (*PtrTUB7*), fragile fibre 1 (*PtrFRA1*), and two myeloblastosis (MYB) genes (*PtrMYB90*, *PtrMYB18*) were examined. *TUA1* (**a**) and *UBQ1* (**b**) were used as reference genes, and the expression level of each gene in the WT background was set to 1. The data are the mean ± standard errors (SE). The asterisks indicate significant differences from WT (*P < 0.05)

Significant changes in the transcript abundance of ten secondary wall-associated transcription genes were observed in *PdRanBP*-OE and *PdRanBP*-DR lines, as determined using qRT-PCR. Five genes associated with cell wall biosynthesis (trans-cinnamate 4-hydroxylase 1 (*PtrC4H1*), cinnamyl alcohol dehydrogenase 10 (*PtrCAD10*), caffeoyl CoA 3-O-methyltransferase 1 (*PtrCCoAOMT1*), glycosyltransferase 8 (*PtrGT8*) and cinnamoyl coenzyme A reductase 7 (*PtrCCR7*)), three gene associated with MFA (sucrose synthase 1 (*PtrSuS1*), beta-tubulin 7 (*PtrTUB7*) and fragile fibre 1 (*PtrFRA1*)), and two myeloblastosis (MYB) genes (MYB; *PtrMYB90*, *PtrMYB18*) were selected for investigation. Compared with WT plants, the expression level of *PtrCCR7* decreased by 80.10 %–82.60 % in the three *PdRanBP*-OE lines (*P* = 0.003, Fig. 6a) and upregulated by 182.5 %–209.40 % in the three *PdRanBP*-DR lines (*P* = 0.004, Fig. 6a). The expression levels of *PtrCCoAOMT1* were significantly decreased by 67.80 %–75.90 %, in the three *PdRanBP*-OE tested lines (*P* = 0.002, Fig. 6a) and significantly upregulated by 109.3 %–314.20 % in the three *PdRanBP*-DR tested lines (*P* = 0.025, Fig. 6a). *PtrFRA1* expression decreased by 57.3 %–78.20 % in the three tested *PdRanBP*-OE lines (*P* = 0.001, Fig. 6a) and increased by 103.9 %–314.20 % in the three tested *PdRanBP*-DR lines (*P* = 0.000, Fig. 6a). The observed patterns of gene expression were the same when using either *TUA1* (Fig. 6a) or *UBQ1* (Fig. 6b) as the control gene. These results indicated that *PdRanBP* might be involved in the regulation of cell wall-related transcription factors/genes and as well as with cell wall biogenesis.

### Transgenic poplar lines increased the proportion of cells in the G2 phase of the cell cycle in poplar

**Fig. 7:**
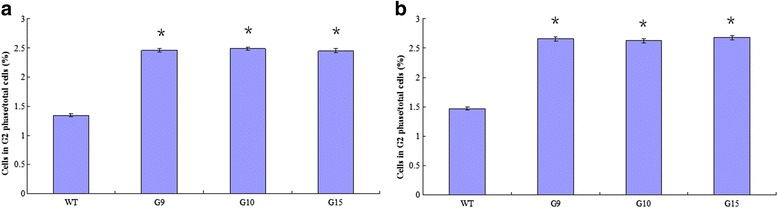
Effect of the *PdRanBP* transgene on the cell cycle phases of poplar cells. **a** The proportion of cells in G2 in the top stems of wild-type (WT) and *PdRanBP*-OE cutting seedlings (G9, G10 and G15). The number of cells in G2 was calculated as a proportion of the total cell population. The results are the mean ± SE from different lines analysed in three independent experiments. **b** The G2 population in the leaf buds of the WT and *PdRanBP*-OE cutting seedlings (G9, G10 and G15). The asterisks indicate significant differences compared with WT (*P < 0.05)

Fluorescence-activated cell sorting (FACS) was used to determine whether the cell cycle was altered in the *PdRanBP* transgenic poplar lines. The average number of G2 phase cells was clearly increased in the stems and leaf buds of the *PdRanBP*-OE lines compared with WT plant cells (1.34 % to 2.47 % in stems, *P* = 0.001; 1.47 % to 2.66 % in buds and *P* = 0.001, Fig. 7). In terms of the cell cycle, these results suggested that *PdRanBP* primarily increased the tendency of cells to remain in G2, thus regulating cell division.

## Discussion

The evaluation of gene expression levels of *PdRanBP* in different organs and tissues in *P. deltoides* has contributed to our understanding of the function of this gene in plant growth. Haizel et al. [32] reported that the *Arabidopsis AtRanBP* genes were expressed in the stems, leaves, roots and flowers, with the highest level of expression being in meristematic tissues, such as the shoot and the root apical meristem. Tian et al. [33] demonstrated that the wheat *TaRanBP* gene is expressed similarly in its stem, leaf and root tissues. Wang et al. [34] found that the transcript levels of the wheat *RAN* gene (*TaRAN1*) were high in young stems and flower buds but low in old leaves. The fescue (*Festuca arundinacea*) Ran GTPase homologous (*FaRan*) gene is broadly expressed in stems, inflorescence meristems, old mature leaves, young leaves and plumules [35]. In this study, high *PdRanBP* expression was observed in in leaf buds, immature xylem and immature phloem. The stem-specific expression pattern of *PdRanBP* in *P. deltoides* is also consistent with the pattern of *AtRanBP* in *Arabidopsis* [32], *TaRanBP* in wheat [33], and *FaRan* in Fescue [35]. This tissue-specific expression pattern indicated that *PdRanBP* might be involved in stem development and wood formation in *P. deltoides*.

The green fluorescent protein (GFP) of the jellyfish *Aequorea victoria* can be visualized directly through emission of green light upon excitation with blue light or long UV [36, 37]. Recently, transient or stable expression of *gfp* has been described in several transformed angiosperm and gymnosperm plants [38, 39]. DNA particle bombardment has been used to produce transgenic soybeans [40], beans [41], peanuts [42], cowpeas [43] and poplars [44, 45]. In this study, the fusion of GFP to the C- terminus of full-length *PdRanBP* resulted in exclusive nuclear labelling in onion epidermal cells and poplar stem cells. This nuclear localization of GFP-tagged PdRanBP was consistent with the expression of the *RanBP* gene in various other plants, such as *Nicotiana benthamiana*, *Oryza sativa* and *Triticum aestivum* [19, 20, 34].

Two types of cell division occur during secondary xylem development: periclinal and anticlinal. Periclinal division determines the number of secondary xylem cells in each radial file, while anticlinal division occurs in the initial cambial cells and determines the number of radial files in the secondary xylem cells [46–49]. To investigate the role of *PdRanBP* in secondary tissues development, we examined transverse sections of the xylem, phloem and cambium regions, which represent the different anatomical features of vascular tissue. The number of cell layers, widths of the vascular tissues (xylem, phloem and cambium), average internode length, and stem height and width were significantly increased in *PdRanBP*-DR plants compared with WT plants. This result is similar to those observed for the *P. deltoides* remorin gene *PdREM* in aspen. The average internode length and the widths of the secondary xylem and secondary phloem and were also increased in *PdREM*-DR lines [50]. Based on these experiments, it appears that *PdRanBP* suppresses cell enlargement directly or indirectly by blocking secondary cell wall synthesis and expansion.

Yeast two-hybrid and co-immunoprecipitation analyses demonstrated the specific interaction of basic helix-loop-helix (bHLH) transcription factors with human RanBP17 [51]. In plants, a bHLH transcription factor was identified as a secondary cell wall regulator that can bind to the promoters of secondary cell wall biosynthesis genes and play an important role in the secondary cell wall regulatory network [52]. bHLH proteins can interact with MYBs [53], and the MYB–bHLH interaction is necessary to control secondary cell wall synthesis in the xylem [54]. In this study, the expression levels of *PtrMYB90* and *PtrMYB18* were significantly decreased (by 67.80 %–75.90 %) in the three tested *PdRanBP*-OE lines (*P* = 0.004 and *P* = 0.003, respectively), In addition, these genes were significantly upregulated (by 109.3 %–314.20 %) in the three tested *PdRanBP*-DR lines (*P* = 0.007 and *P* = 0.011, respectively). We speculated that PdRanBP interacted with MYB, then with MYB–bHLH transcription factors, and ultimately formed protein complexes that induced changes in the expression of secondary cell wall formation-associated genes in poplar.

The cambium is derived from the shoot apical meristem (SAM). Apical regions have common roles in promoting primary growth and accelerate the differentiation of functional cell types. Lu et al. [35] reported that overexpression of the tall fescue *FaRan* gene reduced apical dominance and induced over-proliferation of axillary buds in the rosette leaf axils of transgenic *Arabidopsis*. Wang et al. [19] found that overexpression of wheat (*T. aestivum*) *TaRAN1* increased primordia, delayed flowering, and reduced apical dominance in *Arabidopsis*. In the present study, *PdRanBP* was enriched in the shoot apices (i.e., the stem tip of the 5-cm collected branches) of *PdRanBP*-DR poplar lines (increased secondary wall growth) but not in control and *PdRanBP*-OE lines (Figs. 3 and 4). This result indicated that *PdRanBP* regulates secondary growth via differences in gene expression in stems. *PdRanBP* overexpression induced sylleptic branches and reduced apical dominance, whereas *PdRanBP* downregulation promoted seedling height and shoot growth (Figs. 3 and 4). The apical and radial growth (e.g., stem height and width, and average internode length) of *PdRanBP*-DR lines were greater than in *PdRanBP*-OE lines (Figs. 3 and 4), indicating that *PdRanBP* affects the apical and radial growth of poplar trees.

The MFA is an important property of wood tissues. The angle at which microfibrils are arranged with respect to the longitudinal axis of the cell determines the stiffness of the wood. A high MFAs results in increased longitudinal shrinkage and low wood stiffness. The stiffness of the cell wall increases fivefold as the MFA decreases from approximately 40° to 10° [55]. Thus, a low MFA of wood is a highly undesirable property for the genetic improvement of poplar. MFA is under genetic control [56–58] and can be directly measured in immature trees, providing an attractive option for early selection and trait improvement in poplar. The *P. deltoides* gene *PdCYTOB*, which encodes a cytokinin-binding protein, is related to the wood properties of *P. deltoides*. The MFA of antisense-*PdCYTOB* transformed hybrid poplar (*P. davidiana* × *P. bolleana*) decreased by 4.9 %–24.4 % compared with WT plants in the greenhouse [59]. In a previous study, a 10.0–17.5 % reduction in MFA was observed in *PdREM* antisense-expressing transgenic poplar lines compared with control lines [50]. In the present research, the MFA values of the *PdRanBP*-DR poplar lines GA106, GA515 and GA516 ranged from 10.72° to 11.35°, with a mean of 11.08° and an average SD of 0.045; these differences were statistically significant (*P* = 0.001). All of the transgenic poplar hybrids expressing antisense *PdRanBP* constructs had lower MFAs than the untransformed lines, suggesting that *PdRanBP* gene might play an important role in improving microfibril angles.

Schulze et al. carried out yeast two-hybrid assays, finding that mouse β1-tubulin or β5-tubulin can interact with RanBP10. RanBP10 also interacted with the β5-tubulin isoform in yeast cells, thereby exhibiting nonselective for association with β-tubulins [60]. In plants, Spokevicius showed that a *Eucalyptus grandis* β-tubulin gene (*EgrTUB1*) is involved in determining the orientation of cellulose microfibrils in plant secondary fibre cell walls and that the cellulose microfibril angle (MFA) correlates with *EgrTUB1* expression [61]. In *PdRanBP* transgenic poplar lines, the downregulation of *PdRanBP* significantly increased the expression of *PtrTUB7*, and was associated with a lower MFA. The molecular mechanism by which *PdRanBP* decreases the MFA in transgenic plants is unclear. We hypothesize that poplar PdRanBP may interact with tubulin proteins, such as PtrTUB7, and thereby direct microfibril orientation and determine the MFA in secondary fibre cell walls. Another hypothesis is that *PdRanBP* regulates MFA-associated genes (e.g., *PtrFRA1* and *PtrSuS1*) (Fig. 6); in this way,, downregulation of *PdRanBP* expression would alter the MFA.

The overexpression of *PdRanBP* increased the proportion of cells in the G2 phase. This finding echoes the results of other studies in yeast and rice [19, 34]. Wang et al. [19, 34] found that the average number of cells in G2 increased significantly in *TaRAN1*-transformed yeast or rice cells compared with WT cells. We propose that *PdRanBP*, like many other Ran/RanBPs, regulates cell division during cell cycle progression.

## Conclusions

In conclusion, the cloning and detailed characterization of *PdRanBP* from the developing xylem of poplar trees support the notion that this gene is associated with tree growth and vascular tissue development. *PdRanBP* is predominantly expressed in leaf buds and particular cell types (e.g., immature xylem and immature phloem) within the vascular system. These results indicate that *PdRanBP* is potentially involved in vascular tissue development and wood formation. Full-length PdRanBP-GFP fusion proteins were exclusively observed in the nucleus of onion epidermal cells and poplar stem cells. Using a transgenic approach, we showed that *PdRanBP* might function as a negative regulator in *P. deltoides* to enhance secondary cell wall synthesis and promote cell wall expansion. Further characterization of the wood-associated *PdRanBP* gene will open up new avenues of research that may lead to the optimization of molecular breeding and genetic engineering strategies for improved wood quality.

## Endnotes

All the References within the text were designated using the Endnotes X6 software.

## Methods

### Plant growth conditions and sampling

A 15-year-old *P. deltoides* specimen was used to isolate the *PdRanBP* gene and to analyse its tissue-specific expression pattern. Leaves, leaf buds, stems (immature xylem, mature phloem, immature phloem, mature xylem), and male flower buds were harvested three times from different areas of the plant for the analysis (Additional file 8). The hybrid poplar (*P. davidiana* × *P. bolleana*) is a breed that was developed in China by crossing *P. davidiana* and *P. bolleana* and was used for genetic transformation experiments to characterize *PdRanBP* function.

### Isolation, plant expression vector construction and genetic transformation of PdRanBP

Total RNA was prepared using the RNeasy Plant Mini Kit (Qiagen, Valencia, CA, USA) and cDNA was synthesized using M-MLV reverse transcriptase (Promega, Madison, WI, USA) according to the manufacturer’s instructions. The coding region of *PdRanBP* was amplified from cDNA by reverse transcription PCR (RT-PCR), using the primer pair P1 (Additional file 9), which was designed according to the *PtRanBP6* sequence (accession no. XM_002308612.1). The PCR product was ligated into the pGEM-T Easy vector (Promega) and sequenced; the vector was termed pGEM-T-*PdRanBP*.

The ORF of *PdRanBP* was amplified from pGEM-T-*PdRanBP* using the primers P2 and P3 (Additional file 9), yielding DNA fragments with different restriction sites at the 5′ end and 3′ ends. The amplified DNA constructs were inserted into the *Xba*I and *Sal*I sites of the intermediate vector pGEM-T to yield pGEM-T-sense *PdRanBP* and pGEM-T-antisense *PdRanBP*, respectively. *Xba*I and *Sal*I were used to digest pGEM-T-sense *PdRanBP*, pGEM-T-antisense *PdRanBP*, and the plant expression vector pBI121 (Clontech Labs, Inc., Palo Alto, CA, USA). Lastly, sense and antisense *PdRanBP* constructs were cloned into the pBI121 vector to generate pBI121-sense *PdRanBP* and pBI121-antisense *PdRanBP*, respectively (Additional file 10). The vectors were confirmed by sequencing, separately transformed into *Agrobacterium tumefaciens* (strain GV3101), and subsequently transformed into hybrid poplar (*P. davidiana* × *P. bolleana*) using the leaf disk transformation method [62].

### Characterization of transformed poplars

The transgenic poplar were grown in a greenhouse at the Chinese Academy of Forestry under natural light conditions, with an 18 h light/6 h dark photoperiod at a temperature of 22 °C/15 °C (day/night). Transgenic poplars were identified by PCR using P6 primers (Additional file 9) to amplify the *NptII*-sensitive selective marker gene.

### Analysis of the expression of *PdRanBP* and secondary wall-associated transcription factors/genes by qRT-PCR

qRT-PCR was used to analyse the expression patterns and levels of *PdRanBP* in different tissues of the *P. deltoides* tree and transgenic poplar plants. The expression patterns and levels of secondary cell wall-related genes in 120-day-old *PdRanBP*-OE and *PdRanBP*-DR transgenic plants were also assessed (Additional files 9 and 5). The qRT-PCR analysis was performed using the α-tubulin (*TUA1*) and Ubiquitin (*UBQ1*) gene as internal controls [63], according to the instructions of the SYBR® Premix Ex Taq^TM^ Kit (Takara, Tokyo, Japan). The reactions were run on an ABI Prism 7500 sequence detector (Applied Biosystems, Foster City, CA, USA) using SYBR Green PCR Master Mix (Applied Biosystems). Each PCR reaction (final volume 20 μl) contained 1 μl of first-strand cDNA, 200 nM of primers and 1× SYBR Green PCR Master Mix. Three replicates were conducted in parallel, and statistical analysis of the data was performed following the ABI Prism 7500 Sequence Detection System Users Guide. Gene-specific primer pairs (Additional files 9 and 5) were designed using the software Primer premier 5.0 (Premier Biosoft Int., Palo Alto, CA, USA).

Standard curves were constructed to calculate the gene-specific PCR efficiency from 10-fold series dilutions of the mixed cDNA templates for each primer pair. The correlation coefficients (R^2^) and slope values could be obtained from the standard curve, and the corresponding PCR amplification efficiencies (E) were calculated according to the following equation: E = (10^-1/slope^-1) × 100 [64].

### Construction of the expression vector *EGFP*-*PdRanBP* and plant cell transformation

The enhanced green fluorescent protein (*EGFP*) gene was amplified by PCR from the EGFP vector (Clontech, Palo Alto, CA, USA) using the P5 primer pair (Additional file 9). After digestion of the amplified DNA fragment with *Xba*I, the 715-bp fragment was inserted into the *Xba*I site of pBI121, downstream of the *CaMV* 35S promoter, yielding the *EGFP*-*PdRanBP* vector (Additional file 10). The sequence of the *EGFP*-*PdRanBP* plasmid was confirmed by DNA sequencing, and the vector was transformed into onion cells and poplar (*P. davidiana* × *P. bolleana*) cells using DNA particle bombardment [43, 65].

### *EGFP* fluorescence analysis

To detect fluorescent signals in onion cells transformed with the *EGFP*-*PdRanBP* vector, at least 10 independently transformed lines were observed using an inverted fluorescence microscope (Carl Zeiss, Oberkochen, Germany) with a blue high-sensitivity filter block. The images were captured using a computationally controlled digital camera (AP-1; Apogee Instruments Inc., Tucson, AZ, USA). The images were processed using AxioVision software (Carl Zeiss Inc., Thornwood, NY, USA). The selected sections were processed further using Photoshop 5.0 (Adobe Systems, Mountain View, CA, USA).

### Toluidine blue O staining, DAPI staining and microscopy

Cross sections (approximately 5–10 mm thick) of the internodes of *PdRanBP* transgenic hybrid poplars and WT stems, as well as the tips of *EGFP*-*PdRanBP* transgenic hybrid poplars and WT stems, were fixed overnight at room temperature (RT, 22 °C) in a formalin–alcohol–acetic acid (FAA). The samples were then embedded in paraffin wax, cut into 8-μm sections using a microtome (Leitz, Wetzlar, Germany), and dehydrated through an alcohol series. WT stems and cross sections of the internodes of *PdRanBP* transgenic hybrid poplars were stained with toluidine blue O (TBO), as described by Abbott et al. [66]. WT stems and cross sections of the tips of *EGFP*-*PdRanBP* transgenic hybrid poplars were briefly stained with DAPI (1 mg/mL in mounting medium [Vectashield; Vector Labs, Burlingame, CA, USA]), as described by Jasencakova et al. [67].

The number of radial cell layers and the overall widths of the xylem, phloem and cambium region of *PdRanBP* transgenic hybrid poplar were measured using an inverted fluorescence microscope. The number of nuclei was determined by counterstaining with DAPI (Carl Zeiss). The images were obtained using a digital camera system (AP-1; Apogee Instruments Inc., Tucson, AZ, USA).

### MFA measurement

Blocks of stems were excised 5 cm above ground level from each transgenic and control poplar line. The MFA was determined by the method described by Franklin et al. [68–70]. Briefly, macerated fibres were acquired from the samples by incubation in glacial acetic acid/hydrogen peroxide solution (1:1 *v*/*v*) at 60 °C overnight. Individual fibres were identified on microscope slides, and the MFA was measured by polarized microscopy using an Olympus BX51 microscope (Melville, NY, USA).

### Flow cytometric analysis

The stems and leaf buds of *PdRanBP*-OE transgenic and WT plants were cut with a razor blade. The cells were treated with nuclear isolation buffer [71] and prepared for FACS by staining with propidium iodide (PI) (Annexin-V-FLUOS staining kit, Roche) [72, 73]. Briefly, the cells were fixed in ethanol overnight at 4 °C, washed, and resuspended in 0.4 mL of 30 mM sodium citrate, pH 7.0, containing 0.1 mg/mL RNase A for 2 h at 37 °C. These steps were followed by incubation in 4 mg/mL PI (final concentration). Each sample was analysed using a FACSCalibur flow cytometer (Becton Dickinson, San Jose, CA,USA).

### Phylogenetic analysis and statistical analyses

The *Populus trichocarpa RanBP* sequence (*PtRanBP6*, NCBI accession no. XM_002308612.1) and other RanBP protein sequences were obtained from GenBank. These sequences were then aligned to generate a phylogenetic tree using the MEGA 4.0 software program [74, 75], using the neighbour-joining method.

The growth, wood properties, and all qRT-PCR results were analysed using one-way analysis of variance. Asterisks and/or ‘sig’ indicate significant differences (*P* < 0.05; ANOVA, Fisher test) between the transgenic lines and WT. The statistical analyses were performed using the statistical program SPSS 11.0 (SPSS Inc., Chicago, IL, USA).

## Abbreviations

ABA, Abscisic acid; ANOVA, One way analysis of variance; *AtRanBP1c*, *Arabidopsis RanBP1c* gene; bHLH, basic helix-loop-helix; *C4H*, 4-coumarate:cinnamate-4-hydroxylase; *CAD*, Cinnamyl alcohol dehydrogenase; *CCoAOMT*, Caffeoyl CoA 3-O-methyltransferase; *EgCCR*, *Eucalyptus gunnii* (J.T. Hook) cinnamoyl coenzyme A reductase; EGF, Epidermal growth factor; EGFP, The enhanced green fluorescent protein; *EgrTUB1*, *Eucalyptus grandis* b-tubulin gene; FAA, Formalin–alcohol–acetic acid; FACS, Fluorescence-activated cell sorter; *FaRan*, Fescue (*Festuca arundinacea*) Ran GTPase homologous gene; GFP, Green fluorescent protein; *GhSuSy*, *Gossypium hirsutum* sucrose synthase gene; MFA, Microfibril angle; MYB, Myeloblastoma; *NbRanBP1*, *Nicotiana benthamiana* (Domin.) *RanBP* gene; *OsRAN2*, *Oryza sativa RAN* gene; *PdRanBP*, *P. deltoides* small GTP-binding protein gene; *PdRanBP*-DR, *PdRanBP*-downregulated; *PdRanBP*-OE, *PdRanBP*-overexpressing; PI, Propidium iodide; *PsRan1*, Pea (*Pisum sativum* L., cv. Alaska) *Ran* gene; *PtRanBP6*, *P. trichocarpa RanBP* 6; *PtrCCR7*, *P. trichocarpa* cinnamoyl coenzyme A reductase 7; *PtrFRA1*, *P. trichocarpa* fragile fibre 1; *PtrGT8*, *P. trichocarpa* glycosyltransferase 8; *PtrSuS1*, *P. trichocarpa* sucrose synthase 1; *PtrTUB7*, *P. trichocarpa* beta-tubulin 7; RanBP, Ran-binding protein; SAM, Shoot apical meristem; SND1, Secondary wall-associated NAC domain protein 1; *TaRAN1*, Wheat (*Triticum aestivum* L.) *RAN* gene; TBO, Toluidine blue O; *TUA1*, α-tubulin; *UBQ1*, Ubiquitin; VIGS, Virus-induced gene silencing; VWFC, Von Willebrand factor type C; WT, Wild-type

## Additional files

Additional file 1: The open reading frames (ORFs) of *P. trichocarpa RanBP6* and both *PtRanBP18* and *hpPdRanBP* of hybrid poplar (*P. davidiana* Dode × *P. bolleana* Lauche). (DOC 23 kb) Additional file 2: The amino acid sequences of *P. deltoides* PdRanBP, and the RanBP sequences from other plants. (TXT 6 kb) Additional file 3: Alignment of the deduced amino acid sequences of *P. deltoides* PdRanBP, and the RanBP sequences from other plants. (DOC 2829 kb) Additional file 4: The anatomical features of the stem of the WT and transgenic poplar plants in transverse view. (DOC 36 kb) Additional file 5: Primers used to detect the expression of secondary wall-associated genes by qRT-PCR. (DOC 38 kb) Additional file 6: Melting curves of two reference genes and ten secondary wall-associated genes. (DOC 1689 kb) Additional file 7: Standard curves of two reference genes and ten secondary wall-associated genes. (DOC 152 kb) Additional file 8: Details of the different vascular tissues and other organs sampled from *Populus deltoides*. (DOC 28 kb) Additional file 9: Primers used for gene isolation, plant vector construction and qRT-PCR gene expression analysis in poplar. (DOC 137 kb) Additional file 10: Diagrams of the vectors used for transgenic analysis. (A) Construction of the pBI121-sense *PdRanBP* vector overexpressing the poplar *PdRanBP* gene; (B) Construction of the pBI121-antisense *PdRanBP* vector expressing the antisense poplar *PdRanBP* gene; (C) Construction of the *EGFP*-*PdRanBP* vector expressing the EGFP-PdRanBP fusion protein. (DOC 137 kb)
