# Supplementary material for: Small GTP-binding protein PdRanBP regulates vascular tissue development in poplar
Source: BMC Genet. 2016 Jun 29;17:96. doi: 10.1186/s12863-016-0403-4 (PMC4928302; doi:10.1186/s12863-016-0403-4)
Supplement: Additional file 1: — The open reading frames (ORFs) of P. trichocarpa RanBP6 and both PtRanBP18 and hpPdRanBP of hybrid poplar (P. davidiana Dode × P. bolleana Lauche). (DOC 23 kb) [file 12863_2016_403_MOESM1_ESM.doc]

**Additional file 1：**The open reading frames (ORFs) of *P*. *trichocarpa RanBP6* and both *PtRanBP18* and *hpPdRanBP* of hybrid poplar (*P*. *davidiana* Dode × *P*. *bolleana* Lauche).

> GenBank:XM_002308612.1 (*PtRanBP6* ) CDS
ATGGCTTTGCCGAATCAGCAAACTGTTGATTATCCAAGCTTCAAGCTTGTTATTGTTGGTGATGGTGGCACAGGGAAAACAACATTTGTGAAAAGGCATCTTACTGGCGAGTTCGAGAAGAAATATGAGCCAACTATTGGTGTGGAAGTTCATCCTTTGGATTTCTTCACTAACTGTGGAAAAATTAGATTCTATTGCTGGGATACAGCTGGTCAAGAGAAGTTTGGTGGTCTCAGAGATGGATATTACATCCATGGACAGTGTGCGATCATCATGTTCGATGTTACTGCTCGTTTGACATATAAGAATGTCCCTACATGGCATCGGGATCTTTGCCGGGTTTGTGAGAACATTCCTATTGTTCTTTGTGGAAACAAGGTTGATGTGAAGAACAGGCAGGTGAAGGCAAAGCAAGTCACTTTCCACCGTAAGAAGAACTTGCAGTACTATGAAATTTCAGCAAAGAGCAATTACAACTTTGAGAAGCCCTTCCTGTACCTTGCCAGAAAGCTTGCTGGGGATCCCAATTTGCATTTTGTCGAGTCTCCTGCCCTGGCTCCCCCGGAAGTTACAATTGATCTTGCAGCACAGGCACAGCACGAGGCTGAGCTCGCTGCTGCAGTTAGTCAACCTCTTCCAGATGATGATGATGATGTATTCGATTAA

> GenBank:[XM_002324810.2](http://www.ncbi.nlm.nih.gov/nucleotide/566213646?report=genbank&log$=nucltop&blast_rank=1&RID=948U14JS015) (*PtRanBP18* ) CDS
ATGGCTTTGCCGAATCAGCAAACTGTTGATTATCCAAGCTTCAAGCTTGTAATTGTTGGTGATGGTGGTACAGGAAAGACCACATTCGTTAAGAGGCATCTTACCGGAGAGTTCGAGAAGAAATACGAGCCAACTATTGGTGTGGAAGTGCACCCCTTGGATTTCTTCACTAACTGTGGCAAAATTAGATTCTATTGCTGGGATACAGCTGGTCAAGAGAAGTTTGGTGGTCTTCGAGATGGATACTACATCCATGGTAATTGTGCTATCATCATGTTTGATGTCACTGCTCGGTTGACATACAAGAATGTCCCTACATGGCACAGGGATCTTTGCAGGGTCTGTGAAAACATTCCAATTGTTCTTTGTGGAAACAAGGTGGATGTGAAGAACAGGCAGGTGAAGGCAAAGCAGGTTACGTTTCACAGGAAGAAGAACCTGCAATACTATGAGATTTCAGCTAAGAGCAATTATAATTTTGAGAAGCCATTCTTGTACCTTGCCAGAAAACTTGCTGGGGATCCTAACTTGCATTTTGTTGAGACTCCTGCCTTGGCTCCCCCAGAAGTGCCTATCGACCTTGTAGCCCAAGCACAGCATGAGGCTGAACTTGCTGCTGCTGCTAGTCAACCTCTTCCAGATGATGACGATGATGCATTTGAATAA

> GenBank: KU841447 (*hpPdRanBP*) CDS

ATGGCTTTGCCGAATCAGCAAACTGTTGATTATCCAAGCTTCAAGCTTGTTATAGTTGGTGATGGCGGCACAGGGAAAACAACCTTTGTGAAAAGGCATCTTACTGGAGAGTTTGAGAAGAAATATGAGCCAACTATTGGTGTGGAAGTTCATCCTTTGGATTTCTTCACTAACTGTGGAAAAATTAGATTCTATTGCTGGGATACAGCTGGTCAAGAGAAGTTTGGTGGTCTCAGAGATGGATATTACATCCATGGACAGTGTGCGATCATCATGTTCGATGTTACTGCTCGTTTGACATATAAGAATGTCCCTACATGGCATCGGGATCTTTGCCGGGTTTGTGAGAACATTCCTATTGTTCTTTGTGGAAACAAGGTTGATGTGAAGAACAGGCAGGTGAAGGCAAAGCAGGTCACTTTCCACCGTAAGAAGAACTTGCAGTACTATGAAATTTCAGCAAAGAGCAATTACAACTTTGAGAAGCCCTTCCTGTACCTTGCCAGAAAGCTTGCTGGGGATCCCAATTTGCATTTTGTTGAGTCTCCTGCCCTGGCTCCCCCGGAAGTTACAATTGATCTTGCAGCACAGGCACAGCACGAGGCTGAGCTAGCTGCTGCAGTTAGTCAACCTCTTCCAGATGATGATGATGATGTATTCGATTAA
