# Supplementary material for: Small GTP-binding protein PdRanBP regulates vascular tissue development in poplar
Source: BMC Genet. 2016 Jun 29;17:96. doi: 10.1186/s12863-016-0403-4 (PMC4928302; doi:10.1186/s12863-016-0403-4)
Supplement: Additional file 3: — Alignment of the deduced amino acid sequences of P. deltoides PdRanBP, and the RanBP sequences from other plants. (DOC 2829 kb) [file 12863_2016_403_MOESM3_ESM.doc]

**Additional file 2:** Alignment of the deduced amino acid sequences of *P*. *deltoides* PdRanBP, and the RanBP sequences from other plants.

The GenBank accession number for PtRanBP6: XM_002308612.1; PtRanBP18: [XM_002324810.2](http://www.ncbi.nlm.nih.gov/nucleotide/566213646?report=genbank&log$=nucltop&blast_rank=1&RID=948U14JS015); LjRanBP: Z73959.1; RcRanBP: XM_002515509.1; DlRanBP: JF461291.1; PsRanBP: EF194277.1; VvRanBP: FQ396597.1; NtRanBP: L16787.1; NsRanBP: AY563049.1; CmRanBP: AB015288.1; GmRanBP: AK243772.1; StRanBP: DQ222522.1; CaRanBP: AJ299064.1; SlRanBP: NM_001247091.1; CcRanBP: AB372270.1; AtRanBP: AY116939.1; LeRanBP: L28714.1; HaRanBP: AF495716.1; OsRanBP: AB015288.1; FaRanBP: FJ610236.1; VfRanBP: [Z24678.1](http://www.ncbi.nlm.nih.gov/nucleotide/395071?report=genbank&log$=nucltop&blast_rank=14&RID=RA19KC8401S); hpPdRanBP: KU841447 and PdRanBP: KU841446. “*” is used to indicate the 2Fe-2S ferredoxin-type iron-sulfur binding domain, the “.” means the epidermal growth factor (EGF)-like domain, “**-**” indicates the von Willebrand factor type C (VWFC) domain.

**
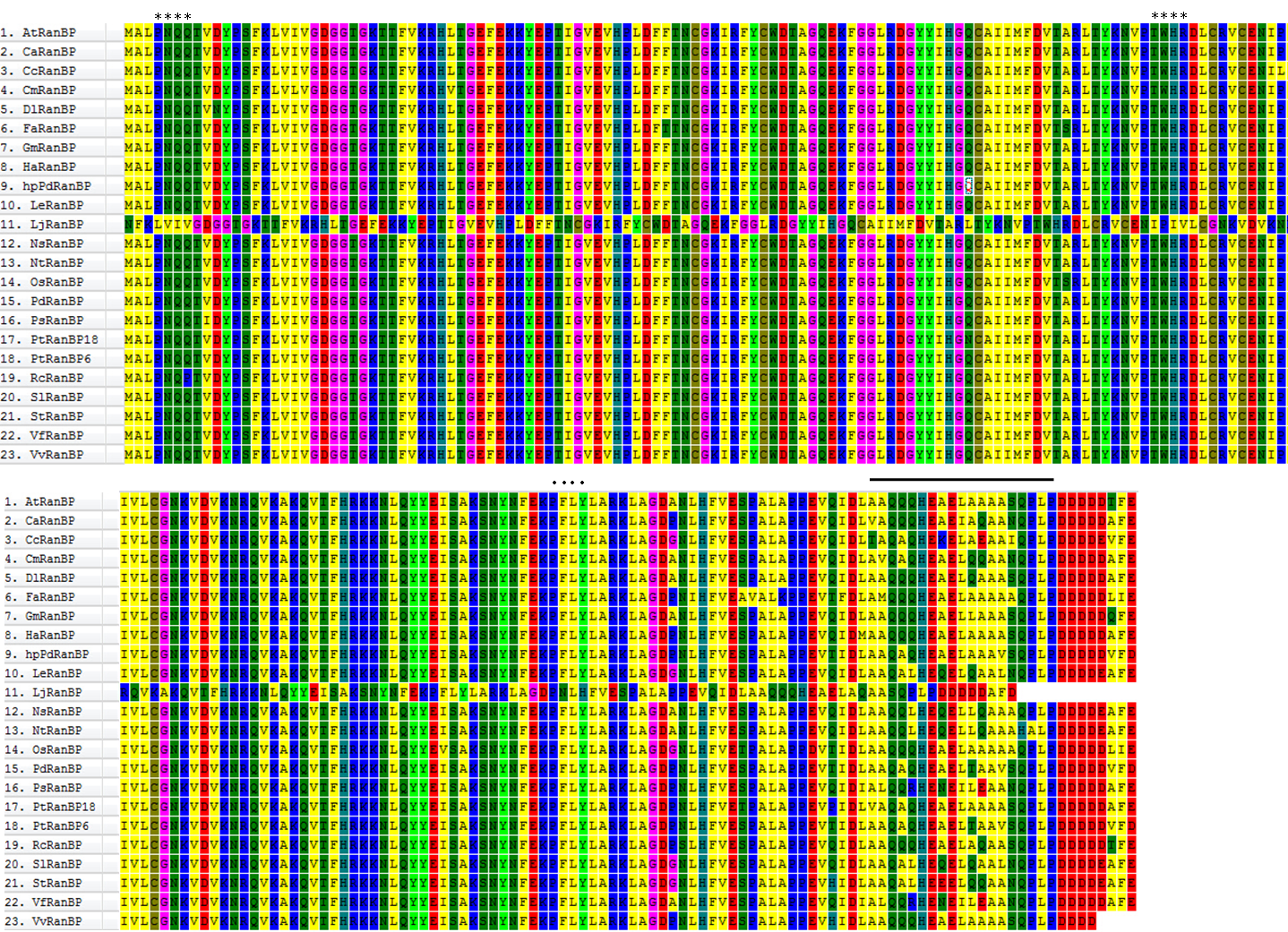
**
