# Supplementary material for: Small GTP-binding protein PdRanBP regulates vascular tissue development in poplar
Source: BMC Genet. 2016 Jun 29;17:96. doi: 10.1186/s12863-016-0403-4 (PMC4928302; doi:10.1186/s12863-016-0403-4)
Supplement: Additional file 4: — The anatomical features of the stem of the WT and transgenic poplar plants in transverse view. (DOC 36 kb) [file 12863_2016_403_MOESM4_ESM.doc]

**Additional file 3:** The anatomical features of the stem of the WT and transgenic poplar plants in transverse view.

| **Poplar line** | **Phloem** | | **Cambium** | | **Xylem** | | **Width ratio of xylem /phloem** |
| --- | --- | --- | --- | --- | --- | --- | --- |
|  | **Number of cell layers** | **Width (µm)** | **Number of cell layers** | **Width (µm)** | **Number of cell layers** | **Width (µm)** |  |
| Non-transgenic poplar | 26-28 | 325.79 | 4–6 | 32.15 | 47-51 | 608.5 | 1.87 |
| *PdRanBP*-OE G9 | 25-27 | 273.18 | 3–5 | 24.63 | 44-47 | 581.35 | 2.13 |
| *PdRanBP*-OE G10 | 24-28 | 298.05 | 4–5 | 27.12 | 44-49 | 574.63 | 1.93 |
| *PdRanBP*-OE G15 | 26-27 | 268.16 | 3–6 | 25.67 | 43-46 | 566.47 | 2.11 |
| *PdRanBP-*DR GA106 | 31-33 | 384.31 | 5–7 | 31.59 | 51-54 | 641.26 | 1.69 |
| *PdRanBP-*DR GA515 | 29-32 | 366.57 | 4–7 | 29.6 | 49-53 | 632.47 | 1.73 |
| *PdRanBP-*DR GA516 | 30-33 | 375.58 | 5–7 | 32.74 | 50-53 | 621.58 | 1.65 |

Notes :

*PdRanBP*-OE, *PdRanBP*-overexpressing lines; *PdRanBP-*DR, *PdRanBP*-

downregulated lines. Cell layers, stems thicken by cell proliferation within the vascular cambium. The vascular cambium increases the number of cell layers in the phloem or xylem zones through periclinal division. The xylem and phloem regions show an orderly radial arrangement, so the cell layers can be accurately counted (see Figure 5A, B, C).
