# Supplementary material for: Small GTP-binding protein PdRanBP regulates vascular tissue development in poplar
Source: BMC Genet. 2016 Jun 29;17:96. doi: 10.1186/s12863-016-0403-4 (PMC4928302; doi:10.1186/s12863-016-0403-4)
Supplement: Additional file 5: — Primers used to detect the expression of secondary wall-associated genes by qRT-PCR. (DOC 38 kb) [file 12863_2016_403_MOESM5_ESM.doc]

**Additional file 7:** Primers used to detect the expression of secondary wall-associated genes by qRT-PCR.

| **Gene name** | Description | NCBI or JCI accession number | Primer sequence  (forword/reverse) | Amplification  efficiency (%) | R^2^ |
| --- | --- | --- | --- | --- | --- |
| *PtrC4H1* | 4-coumarate:cinnamate-4-hydroxylase | AF302495.1 | CCCTCTTGGGTTCTTTCGTT/CAAACACGGGGACAGGTATA | 99.07 | 0.9933 |
| *PtrCAD10* | Cinnamyl alcohol dehydrogenase | XM_002300175.1 | CAGCACTTTGTACTCCGTATTCC/TGCTTCCCTGGTTCTGTCATT | 95.89 | 0.9967 |
| *PtrCCoAOMT1* | Caffeoyl CoA 3-O-methyltransferase | XM_002313089 | TTGGTGGGCTGATTGGGTA/  GCTCCAAAACAAAGTCCCTGT | 100.005 | 0.9961 |
| *PtrGT8* | Glycosyltransferase 8 | XM_002302433 | AAGCCGAACGAATGTGCCT/  CTCTTCAACCCAGTGATGGT | 91.29 | 0.9995 |
| *PtrCCR7* | Cinnamoyl coenzymeA reductase | XM_002303809.1 | GGCTAAGGAGAAAGGGGTGG/GCCGGTGAGGTACTTGAGGA | 97.67 | 0.997 |
| *PtrSuS1* | Sucrose synthase 1 | GU559729.1 | TTTCCCTCGCCCAACTCTT/  GATGCAGGCTTTCCTTGTCA | 94.55 | 0.9994 |
| *PtrTUB7* | beta-tubulin 7 | XM_002299006.1 | TTGAGCCATACAACGCCAC /  CGGAAGCAGATGTCATACAAA | 98.61 | 0.9972 |
| *PtrMYB90* | Myeloblastoma 90 | XM_002329715 | CCGAAGAGGAAGAAGAAAGACT/TCAGTGCGACCTGGGAAAA | 95.85 | 0.9972 |
| *PtrMYB18* | Myeloblastoma 18 | XM_00230517 | TCATGCTCAACTTGGCAACAG/CAATTCCCATCTTCCTTAGCTT | 91.87 | 0.9995 |
| *PtrFRA1* | Fragile fiber 1 | XM_002302396.1 | CAGCAGAACTGTTATGATAGC / TGTTGCGGGCACGATTTG | 94.13 | 0.9973 |
| *TUA1* | α-tubulin 1 | CA822230;CA825391 | AGGTTCTGGTTTGGGGTCTT/TTGTCCAAAAGCACAGCAAC | 97.7 | 0.9985 |
| *UBQ1* | Ubiquitin 1 | BU879229 | GTTGATTTTTGCTGGGAAGC/GATCTTGGCCTTCACGTTGT | 95.92 | 0.9977 |
