# Supplementary material for: Small GTP-binding protein PdRanBP regulates vascular tissue development in poplar
Source: BMC Genet. 2016 Jun 29;17:96. doi: 10.1186/s12863-016-0403-4 (PMC4928302; doi:10.1186/s12863-016-0403-4)
Supplement: Additional file 6: — Melting curves of two reference genes and ten secondary wall-associated genes. (DOC 1689 kb) [file 12863_2016_403_MOESM6_ESM.doc]

**Additional file 8:** Melting curves of two reference genes and ten secondary wall-associated genes. Melting curves of 12 tested genes shows single peaks.


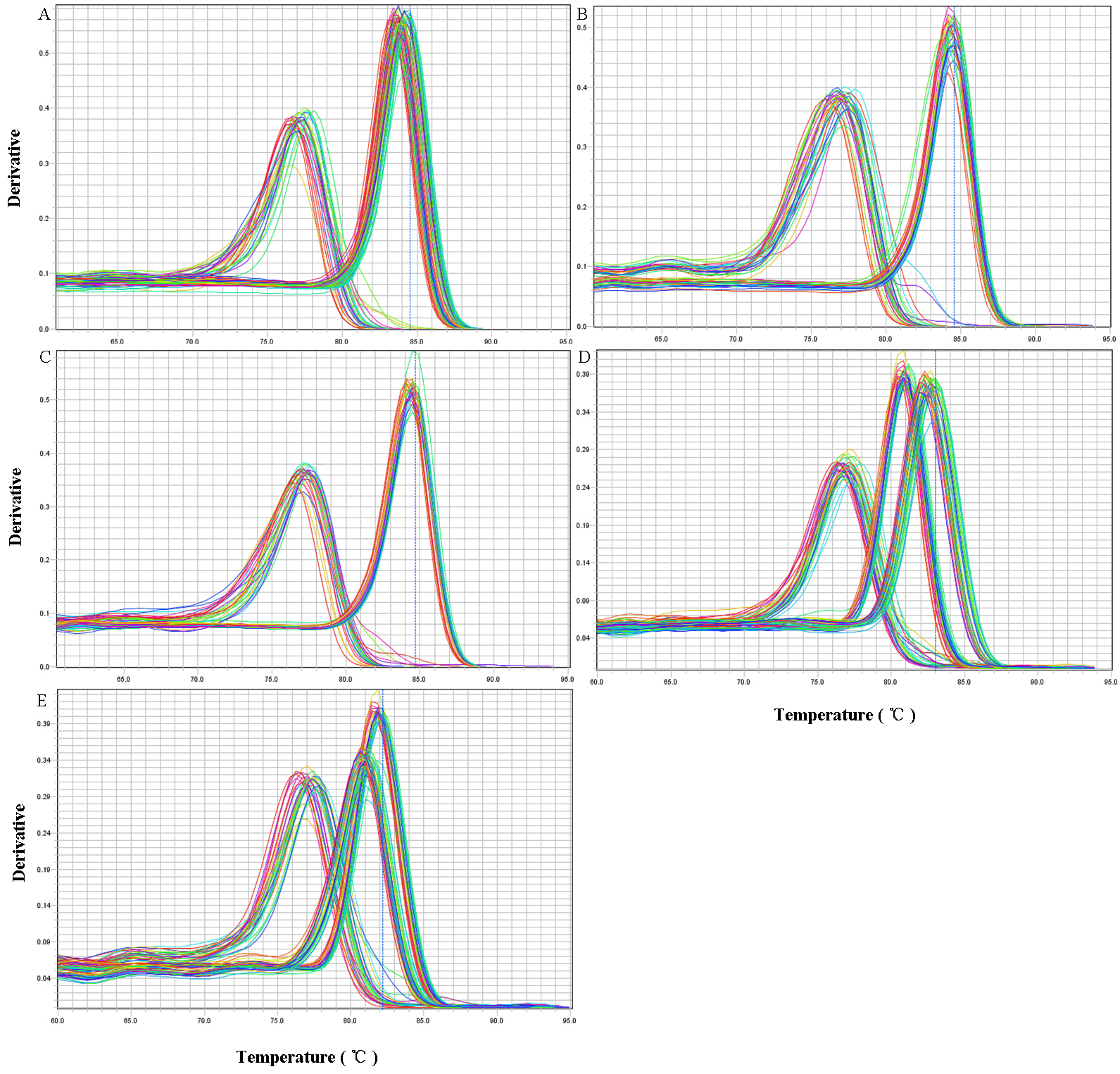


The left and right melting curves of figure A represents the tested genes *PtrGT8* and *PtrMYB90*, respectively.

The left and right melting curves of figure B represents the tested genes *PtrTUB7* and *PtrCCR7*, respectively.

The left and right melting curves of figure C represents the tested genes *TUA1* and *PtrCAD10*, respectively.

The left, middle and right melting curves of figure D represents the tested genes *UBQ1*, *PtrSuS1* and *PtrMYB18*, respectively.

The left, middle and right melting curves of figure E represents the tested genes *PtrFRA1*, *PtrC4H1* and *PtrCCoAOMT1*, respectively.
