# Supplementary material for: Small GTP-binding protein PdRanBP regulates vascular tissue development in poplar
Source: BMC Genet. 2016 Jun 29;17:96. doi: 10.1186/s12863-016-0403-4 (PMC4928302; doi:10.1186/s12863-016-0403-4)
Supplement: Additional file 7: — Standard curves of two reference genes and ten secondary wall-associated genes. (DOC 152 kb) [file 12863_2016_403_MOESM7_ESM.doc]

**Additional file 9:** Standard curves of two reference genes and ten secondary wall-associated genes. The correlation coefficients (R^2^) can be obtained from the standard curve，and PCR amplification efficiencies (E) can be calculated according to the equation E= (10-1/slope-1)×100 [64].


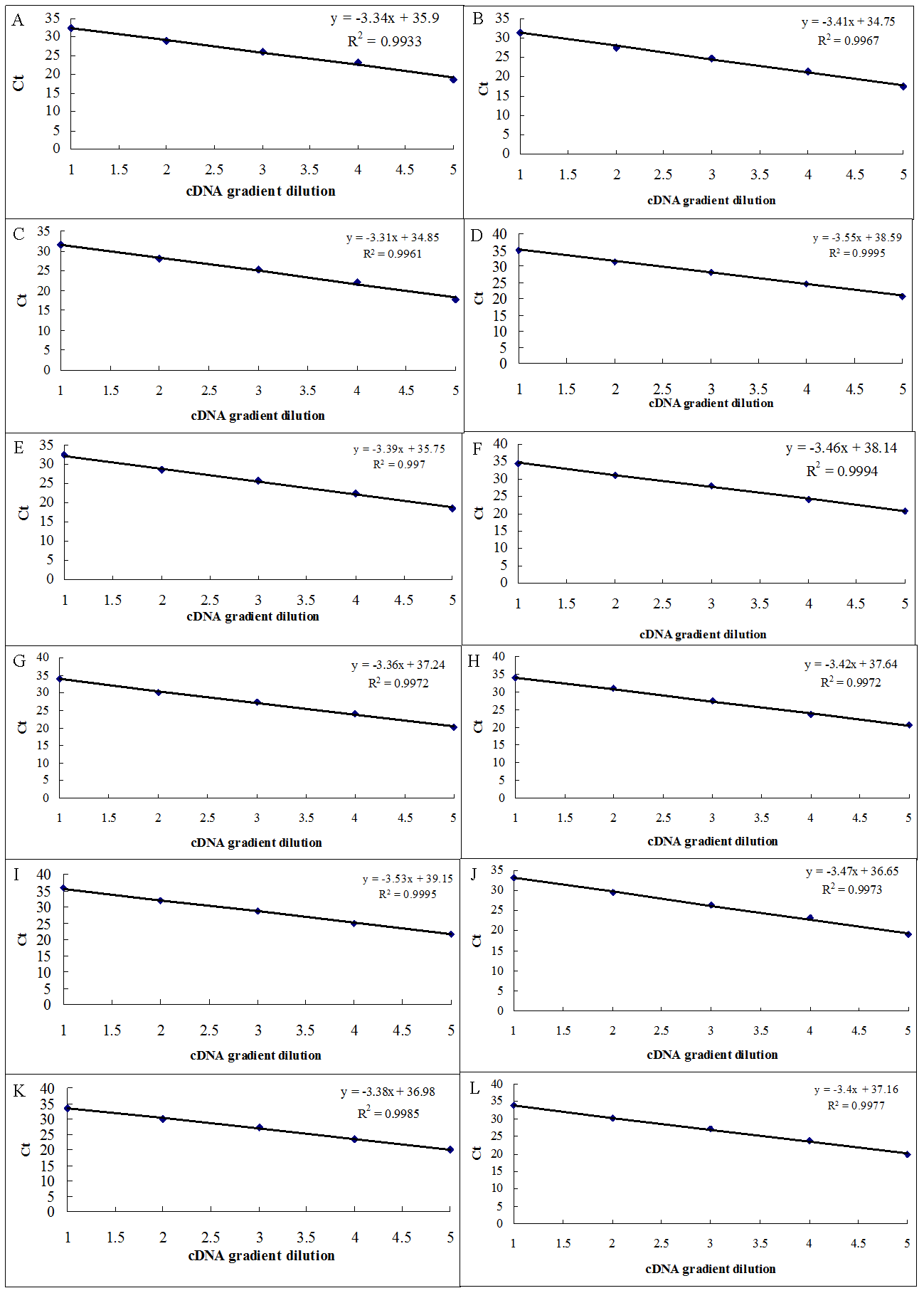


Figure A shows the standard curve of *PtrC4H1*.

Figure B shows the standard curve of *PtrCAD10*.

Figure C shows the standard curve of *PtrCCoAOMT1*.

Figure D shows the standard curve of *PtrGT8*.

Figure D shows the standard curve of *PtrCCR7*.

Figure F shows the standard curve of *PtrSuS1*.

Figure G shows the standard curve of *PtrTUB7*.

Figure H shows the standard curve of *PtrMYB90*.

Figure I shows the standard curve of *PtrMYB18*.

Figure J shows the standard curve of *PtrFRA1*.

Figure K shows the standard curve of *TUA1*.

Figure L shows the standard curve of *UBQ1*.
