# Supplementary material for: Small GTP-binding protein PdRanBP regulates vascular tissue development in poplar
Source: BMC Genet. 2016 Jun 29;17:96. doi: 10.1186/s12863-016-0403-4 (PMC4928302; doi:10.1186/s12863-016-0403-4)
Supplement: Additional file 8: — Details of the different vascular tissues and other organs sampled from Populus deltoides. (DOC 28 kb) [file 12863_2016_403_MOESM8_ESM.doc]

**Additional file 4:** Details of the different vascular tissues and other organs sampled from *Populus deltoides*.

| **Name** | **Description** | **Collection Date** |
| --- | --- | --- |
| **Immature xylem** | Collected from a vigorously developing *Populus deltoides* Marsh.tree in a field near the Chinese Academy of Forestry. Bark was peeled and four- to eight-cell layers which were partially lignified were shaved off from the wood side at chest height. Samples were scraped into liquid nitrogen. | 2009/8/20 |
| **Immature phloem** | The bark was peeled and four- to eight-cell layers from the bark side, representing immature phloem were scraped into liquid nitrogen. | 2009/8/20 |
| **Mature phloem** | Isolated from the bark side, nearer to the pith than the immature phloem. The sample was stiffer than immature phloem. Samples were scraped into liquid nitrogen. | 2009/8/20 |
| **Mature xylem** | Isolated from the exposed wood core, closer to the pith. It was the most rigid vascular tissue. Samples were scraped into liquid nitrogen. | 2009/8/20 |
| **Leaf bud** | Very short internodes of the central branches (not apex), not the developmental branches. They initiate from leaf primordia and form branches and mature leaves after coming into bloom. | 2009/8/20 |
| **Male flower bud** | The terminal buds of shoot apex/tip of a 15-year-old adult male tree. Prerequisite for floral/flower initiation or differentiation during the growth stage. | 2009/8/20 |
