# Supplementary material for: Small GTP-binding protein PdRanBP regulates vascular tissue development in poplar
Source: BMC Genet. 2016 Jun 29;17:96. doi: 10.1186/s12863-016-0403-4 (PMC4928302; doi:10.1186/s12863-016-0403-4)
Supplement: Additional file 9: — Primers used for gene isolation, plant vector construction and qRT-PCR gene expression analysis in poplar. (DOC 137 kb) [file 12863_2016_403_MOESM9_ESM.doc]

**Additional file 5:** Primers used for gene isolation, plant vector construction and qRT-PCR gene expression analysis in poplar.

| **Primer name** | **Forward primer (**5′-3′**)** | **Reverse primer (**5′-3′**)** |
| --- | --- | --- |
| P1 | ATGGCTTTGCCGAATCAGCA | TTAATCGAATACATCATCATCATC |
| P2 | GCCTCTAGAATGGCTTTGCCGAATCAGCA | GGCCAGCTGTTAATCGAATACATCATCATCATC |
| P3 | GCCCAGCTGATGGCTTTGCCGAATCAGCA | GGCTCTAGATTAATCGAATACATCATCATCATC |
| P4 | TCCGGCCGCTTGGGTGGAGAG | CTGGCGCGAGCCCCTGATGCT |
| P5 | TCGACGTTACTGCTCGTTTGAC | GGTACAGGAAGGGCTTCTCAAA |
| P6 | AGGTTCTGGTTTGGGGTCTT | TTGTCCAAAAGCACAGCAAC |
| P7 | GTTGATTTTTGCTGGGAAGC | GATCTTGGCCTTCACGTTGT |

Notes :

(1) Primer pair of P1 for amplifing the coding region of *PdRanBP* from cDNA.

(2) *Xba*I and *Sac*I sites were introduced as underlined in P2, respectively;

(3) *Sac*I and *Xba*I sites were introduced as underlined in P3, respectively;

(4) qRT-PCR primers P4 for *NptII* gene, P5 for *PdRanBP*, P6 and P7 for *TUA1* and *UBQ1*.
