# Supplementary material for: Small GTP-binding protein PdRanBP regulates vascular tissue development in poplar
Source: BMC Genet. 2016 Jun 29;17:96. doi: 10.1186/s12863-016-0403-4 (PMC4928302; doi:10.1186/s12863-016-0403-4)

**Additional file 6:** Diagrams of the vectors used for transgenic analysis. (A) Construction of the pBI121-sense *PdRanBP* vector overexpressing the poplar *PdRanBP* gene; (B) Construction of the pBI121-antisense *PdRanBP* vector expressing the antisense poplar *PdRanBP* gene; (C) Construction of the *EGFP*-*PdRanBP* vector expressing the EGFP-PdRanBP fusion protein.


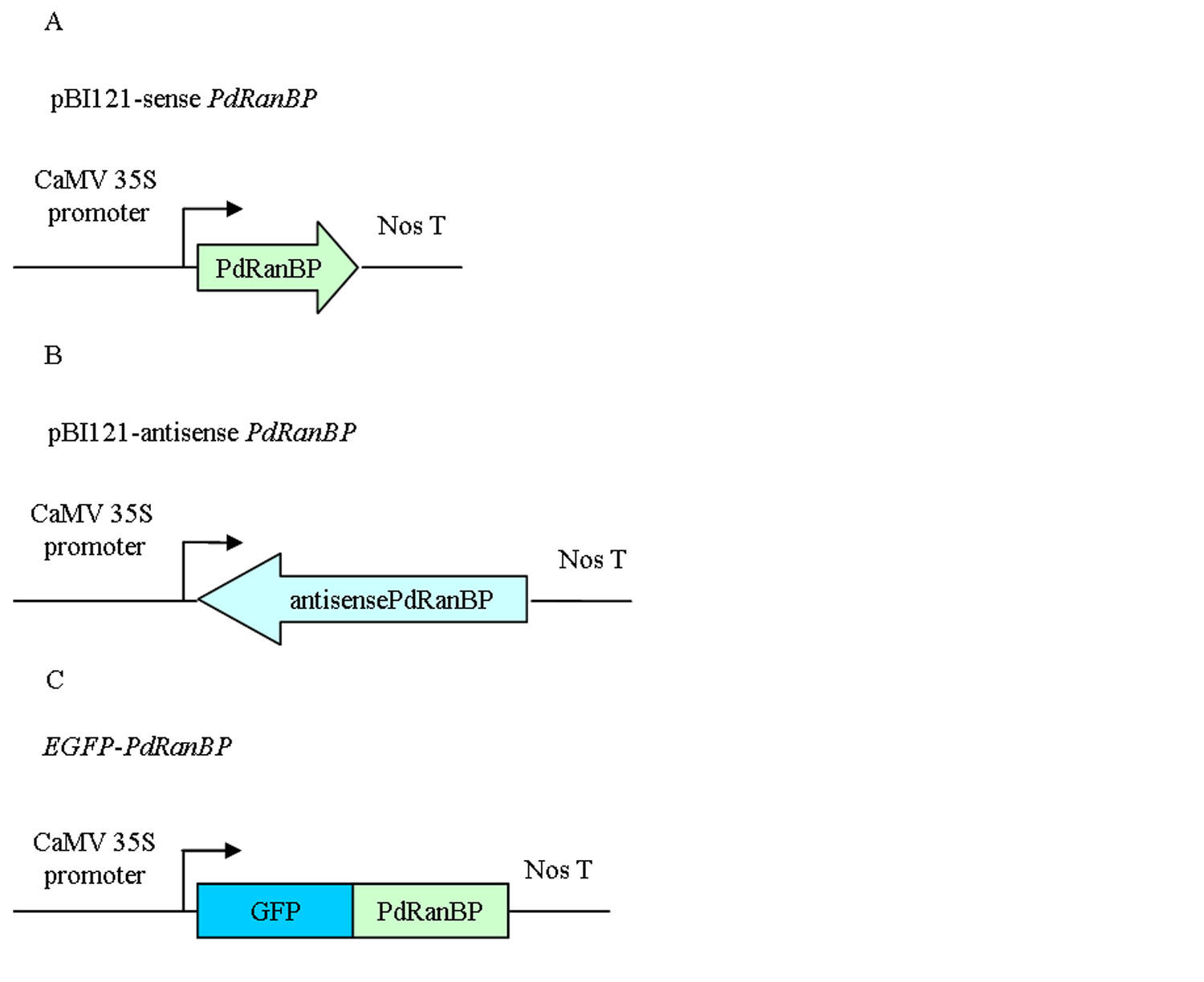

Supplement: Additional file 10: — Diagrams of the vectors used for transgenic analysis. (A) Construction of the pBI121-sense PdRanBP vector overexpressing the poplar PdRanBP gene; (B) Construction of the pBI121-antisense PdRanBP vector expressing the antisense poplar PdRanBP gene; (C) Construction of the EGFP-PdRanBP vector expressing the EGFP-PdRanBP fusion protein. (DOC 137 kb) [file 12863_2016_403_MOESM10_ESM.doc]
